# A model using mutual influence of firing rates of corticomotoneurons for learning a precision grip task

**DOI:** 10.1186/1471-2202-12-S1-P95

**Published:** 2011-07-18

**Authors:** Octave Boussaton, Laurent Bougrain, Thierry Vieville, Selim Eskiizmirliler

**Affiliations:** 1 CORTEX team-project, Nancy University/LORIA/INRIA Nancy Grand Est, Campus Scientifique - BP 239 - 54506 Vandoeuvre-lès-Nancy Cedex, France; 2 CESEM - CNRS UMR 8194, Université Paris Descartes, 45 rue des Saints-Pères, 75270 Paris, France

## 

As a part of a Brain-Machine Interface, we are currently defining a model for learning and forecasting muscular activity, given sparse brain activity in the form of action potential signals (spike trains). We have been working on the flexion of a finger during a trained precision grip performed by a monkey (*macaca nemestrina*), as she clasps a metal gauge with her finger and thumb. Experimentally, the activity of about a hundred neurons in the motor cortex can be recorded simultaneously with the help of a multielectrode array, see Figure **A** and [1] for more details about retrieving and filtering the data. Our method is based on a system of equations involving the firing rate of each recorded neurons, a set of thresholds, and Euclidian distances between averaged and current state at each time step. The firing rates are computed according to given time-windows, between 25 and 100 milliseconds. The thresholds used depend on these firing rates. The learning is done on a subset of the experiments and then evaluated on what remains. A raw estimation is done in order to be used as a reference for estimating the efficiency of each part of the learning formula brings to the final result. The complete improvement formula is divided into three stages and can be expressed as follows: p(t+1) = p(t) + A(t) + B(t) + C(t) where p(t) is the force exerted on the gauge at time t. The A part is the learning reference base of the method in which a straight matching is done between each neuronal code and the derivative of the observed force in the finger at each timestep. What we call a neuronal code is the vector of all the values of the firing rate functions at any given time and during the training stage, to any neuronal code is associated the average value of all the recorded derivatives of the force. The B part is an actuation made on the distance between the current activity of each neuron and its average activity over a former time window of the same length as the time-window used to compute the spike trains. Finally, the C part is a system of equations in which we suppose that every neuron is correlated to each other in a weighted way we optimize during the learning process. The purpose of this study is multifold, we want to estimate (i) the influence of the neurons on each other qualitatively, (ii) the efficiency of various easy-to-tract improvement techniques and (iii) the importance of thresholding the firing rates.

**Figure 1:**
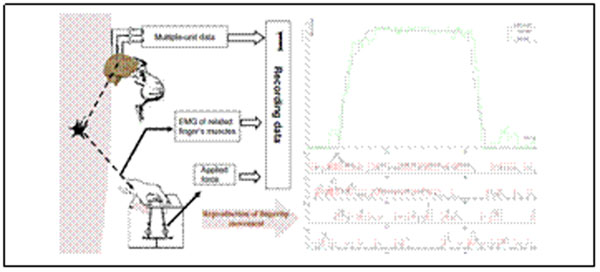
On the left is depicted the experiment. On the right is an example of what can be obtained through our method.The black curve is the observed trajectory that the gray one is supposed to approximate. In this case we used the information of four neurons to tune the parameters of the learning formula and 10 experiments in the learning set. The size of the time window was 80 milliseconds.

We are developing a kind of a systematic approach to spike trains analysis and how they are related to the execution of a movement that allows us to better estimate the influence of several factors, without separating neurons into different groups initially, as in [3] for example but rather consider the information as a whole. The pre-treatment phase ensures that any measurement (corresponding to what we earlier called a neuronal code) is as relevant as any other. The results are quite satisfying and encouraging, given the very restricted complexity of the method, see Figure 1A.
